# Small peptide formulas versus standard polymeric formulas in critically ill patients with acute gastrointestinal injury: a systematic review and meta-analysis

**DOI:** 10.1038/s41598-023-47422-z

**Published:** 2023-11-22

**Authors:** Youquan Wang, Yanhua Li, Hongxiang Li, Yuting Li, Xinyu Li, Dong Zhang

**Affiliations:** Department of Critical Care Medicine, The First Hospital of Jilin University, Chaoyang District, Changchun City, 130021 Jilin Province China

**Keywords:** Gastroenterology, Medical research

## Abstract

Small peptide formulas versus standard polymeric formulas for enteral nutrition in critically ill patients with acute gastrointestinal injury (AGI) have been a topic of debate. A systematic review and meta-analysis were conducted to compare their clinical and nutritional outcomes. Relevant studies from January 1980 to June 2022 were searched in PubMed, Cochrane, and Embase databases. Randomized controlled trials involving AGI grade I-IV patients were included, while children, non-AGI patients, and non-critically ill patients were excluded. Results indicated no significant difference in all-cause mortality. Patients receiving small peptide formulas showed higher daily protein intake, greater albumin growth, and higher prealbumin levels. They also had shorter lengths of stay in the intensive care unit and hospital. Conversely, patients receiving standard polymeric formulas had a higher daily calorie intake. In conclusion, the choice of formula may not affect mortality in critically ill patients with AGI. Small peptide formulas were more conducive to increase daily protein intake, decrease intensive care unit and hospital length of stay. Further large-scale randomized controlled trials evaluating the effects of these two nutritional formulas on clinical and nutritional outcomes in critically ill patients with AGI are needed to confirm these results.

## Introduction

The gastrointestinal tract is a vital organ that plays a key role in nutrient digestion, absorption, and assimilation^1^. It is vulnerable for critically ill patients since intestinal integrity is impaired by reduced epithelial cell proliferation, mucous integrity, increased epithelial cell apoptosis, and permeability^2^. The Working Group on Abdominal Problems of the European Society of Intensive Care Medicine proposed a set of definitions and different grades for gastrointestinal dysfunction. They defined acute gastrointestinal injury (AGI) as a malfunction of the gastrointestinal tract in critically ill patients due to acute illness^3^. Studies have shown that the prevalence of AGI in critically ill patients was 40%, and the mortality of critical patients with AGI is higher than that of patients without AGI^4–6^, what’s more, the mortality rate increases with AGI grades^7^. However, the monitoring of gastrointestinal function is limited and overlooked. To improve clinical outcomes, we should pay sufficient attention to reducing gastrointestinal injury in critically ill patients^1^.

Adequate nutritional support is crucial for restoring cells, organ function, and wound healing in critically ill patients^8^, especially those with AGI. EN can reduce the incidence of infection, postoperative complications, and the length of hospital stay^9,10^, maintain small intestinal structure and function^11^, and promote the restoration of enterocyte mass and create a more optimal microbiota profile compared with parenteral nutrition^12–14^. Nevertheless, AGI patients have digestive and absorption disorders, impaired mucosal barrier function, intestinal flora migration, and increased intestinal vascular permeability^3,15–17^. Therefore, feeding intolerance syndrome often happens in patients with AGI, which may lead to a reduction or even interruption of EN feeding, resulting in malnutrition, and may even affect clinical outcomes^18–21^. EN feeding is a double-edged sword for patients with AGI, and it is particularly important to develop a reasonable EN feeding strategy.

Choosing appropriate nutrition formulas is an important part of the EN strategy for AGI patients, which can fully use the advantages of EN. In theory, small peptide formulas have advantages over standard polymer formulas, including increased gastrointestinal tolerance, accelerated gastric emptying, and reduced incidence of diarrhea. Studies have shown that the transport of the bacterial product N-formyl-methionyl-leucyl-phenylalanine (fMLP) in rat colon increases the expression of oligopeptide transporter (PepT1)^22,23^, which may lead to colonic mucosa damage, the small peptide has competitive inhibition of fMLP transport or greater efficiency of transportation to reduce the expression of PepT1, thus playing a role in intestinal protection^24^. The advantage of the standard polymer formulas are that they are closer to the physiological conditions of the human body, which can promote the secretion of digestive enzymes, and the slow rate of absorption can also promote protein deposition after meals^25^. Society of Critical Care Medicine (SCCM) and the American Society for Parenteral and EN suggest considering the use of small peptide formulations in patients with persistent diarrhea, suspected malabsorption, ischemia, or lack of response to fiber^26^. However, evidence for this recommendation is of low quality and highly subjective, and randomized controlled trials (RCTs) have been documented inconsistently and are very controversial. Therefore, we conducted a meta-analysis, which extracted results from published RCTs to compare the nutritional and clinical outcomes of small peptide formulas and standard polymeric formulas for critically ill patients with AGI.

## Methods

### Protocol and registration

This systematic review and meta-analysis are reported according to the Preferred Reporting Items for Systematic Reviews and Meta-Analyses (PRISMA) guidelines^27^ (Additional file 1: Table S1). The protocol for this meta-analysis is available in PROSPERO (CRD42022332185. Registered 10 May 2022).

### Eligibility criteria

Only RCTs were included in this review, and participants were critically ill patients with AGI grade I-IV. Inclusion and exclusion criteria are outlined in Table 1.

**Table 1:** Criteria to choose studies for the review based on the Population, Intervention, Comparator, Outcomes and Study designs (PICOS) Structure.

	Inclusion criteria	Exclusion criteria
Population	Critically ill patients ≥ 16 years of age with AGI grade I-IV	Children
		Patients without AGI
		Non-critically ill patients
Intervention	Small peptide formulas (Feeding is done through a nasogastric or jejunal tube)	Additional adjuvant therapy (Probiotics, immunomodulating diet or glutamine etc.) was given compared with the control group
Comparator	Standard polymeric formulas (Feeding is done through a nasogastric or jejunal tube)	Nutritional strategies (except type of formula) or interventions differed from the experimental group
Outcomes	Clinical and nutritional outcomes ^a^	Subjective outcomes
Study Design	Randomized controlled trial	Letters, reviews, comments, retrospective, crossover or observational study

AGI: acute gastrointestinal injury.

^a^Clinical outcomes such as mortality, length of stay, etc. Nutritional outcomes such as feeding tolerance, caloric intake and protein intake.

### AGI criteria

The 2012 ESICM guidelines^3^ proposed the definition of AGI (the malfunctioning of the gastrointestinal tract in critically ill patients due to their acute illness, additional file: Table S2). They proposed the concepts of primary AGI (associated with a primary disease or direct injury to organs of the gastrointestinal system) and secondary AGI (the consequence of a host response in critical illness without primary pathology in the gastrointestinal system) depending on the cause of AGI. The scale of RCTs using AGI to define patient populations is modest (possible reasons include: trials conducted before 2012, patients without AGI, patients with AGI but without gastrointestinal function assessment, etc.). The full text was screened to assess the study participants’ primary disease, occurrence, and risk of gastrointestinal adverse events. Studies that did not meet AGI criteria were excluded. Considering that the different sources of gastrointestinal injury (first or second hit^3^) may have different impacts on clinical outcomes, nutritional outcomes, and gastrointestinal adverse events in critically ill patients, we conducted subgroup analysis as follows:

Only secondary AGI: All patients included in the study only had secondary AGI.

Primary and secondary AGI: Some enrolled patients had primary AGI, and the rest had secondary AGI.

### Data sources and search strategies

We comprehensively searched articles and references in the PubMed, Embase, and Cochrane Library databases. The literature search was carried out, without language restrictions, from 01 January 1980 to 30 May 2022. The search was slightly adjusted according to the requirements of the different databases. The search strategy (Additional file 1: Table S3) was designed and formulated by the librarians of our college. Two researchers (YQ Wang and YH Li) selected the studies independently; they identified relevant articles by browsing titles and abstracts and then read the full text to decide whether to include them. Any dispute was solved through discussion by a team of other researchers.

### Types of outcome measures

The primary outcome was all-cause mortality (28-day mortality, 90-day mortality, and hospital mortality).

The secondary outcomes were daily calorie and protein intake, serum levels of albumin and prealbumin, nitrogen balance, gastrointestinal adverse events (including diarrhea, gastric retention > 500 ml, and vomiting), ICU length of stay, hospital length of stay, infections, and mechanical ventilation duration. Weighted means were calculated based on the number of patients in each study.

### Quality assessment

**Figure 1:**
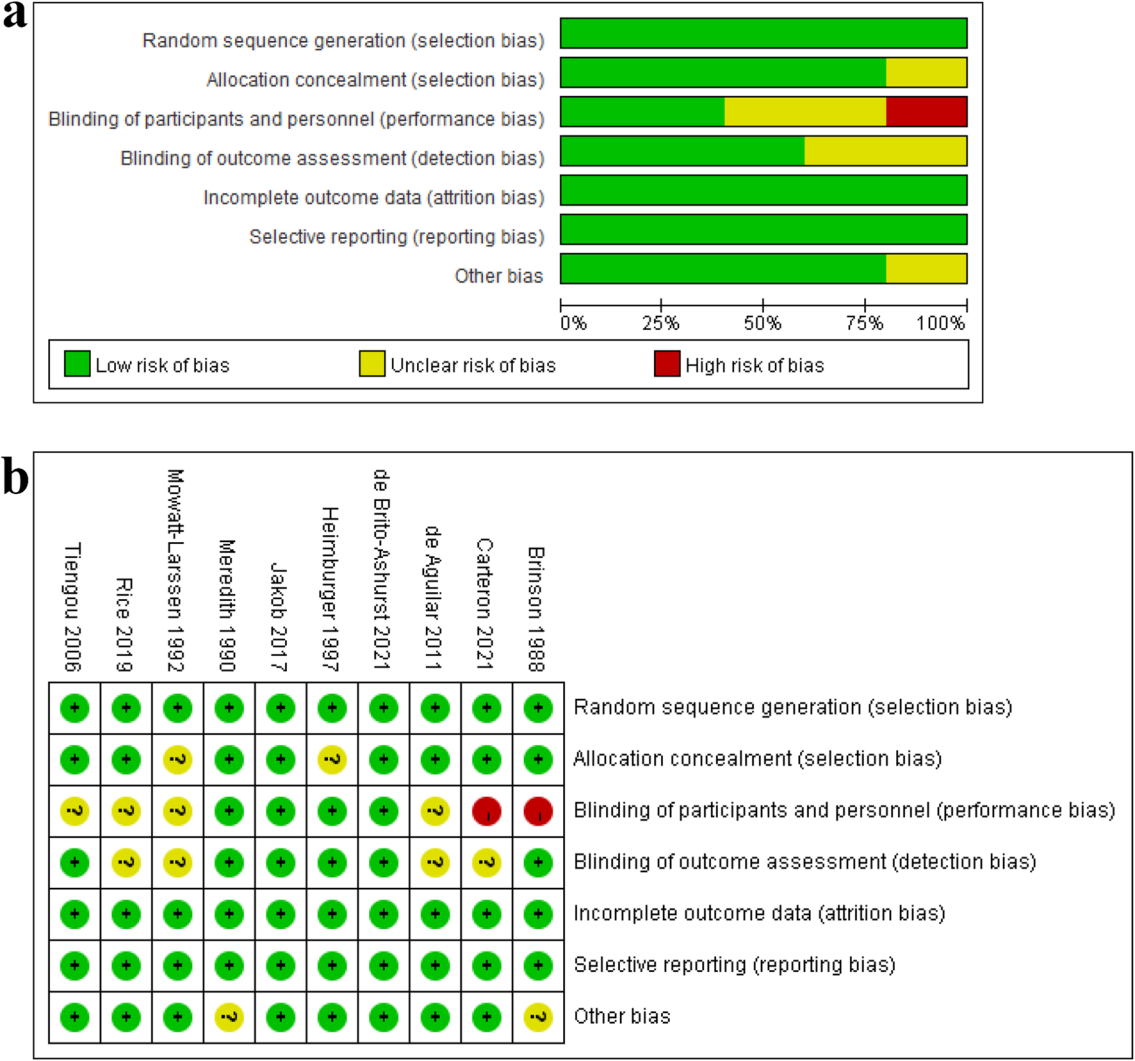
Risk of bias graph: review authors’ judgements about each risk of bias item presented as percentages across all included studies (**a**). Risk of bias summary graph: review authors’ judgements about each risk of bias item for each included study (**b**).

Two reviewers (YQ Wang and YH Li) independently used the Cochrane risk assessment tool to assess the methodological quality of the included trials^28^. The specific elements were adequacy of the methods used to minimize bias through (1) randomization sequence (selection bias), (2) allocation concealment (selection bias), (3) blinding of study personnel and participants (performance bias), (4) blinding of outcome assessors (performance bias), (5) complete reporting of data without arbitrarily excluded patients and with low to minimal loss to follow-up (attrition bias), (6) selective reporting bias, and (7) other sources of bias. Satisfactory performance, unclear performance, and unsatisfactory performance of each domain from the tool are denoted by green, yellow, and red colours, respectively. Disagreements were solved by a discussion with a third author (Hongxiang Li or D Zhang). The risk of bias summary and graph are presented in Fig. 1.

### Statistical analysis

Articles that met the inclusion criteria and did not meet the exclusion criteria for meta-analysis were exported to Review Manager Version 5.3 (RevMan, The Cochrane Collaboration, Oxford, UK) for data analysis. Relative risk (*RR*) with 95% confidence intervals (*CI*) was calculated for dichotomous variables. As to the continuous variables, mean difference (*MD*) and 95% CI was estimated as the effect result. A random-effects model was used to pool studies with significant heterogeneity, as determined by the chi-squared test (*P* < 0.10) and inconsistency index (*I*^*2*^ ≥ 50%)^29^. Some of the selected continuous variables were represented by the median (interquartile range). We calculated their mean and standard deviation according to the sample size with a calculator^30^ and then performed a meta-analysis. A sensitivity analysis was conducted to test the robustness and reliability of all outcomes. Engage Digitizer was used to extract data points from images of graphs. Funnel plot and *Egger’s* or *Begg’s* weighted regression tests were applied to identify any potential publication bias and a *P* value of < 0.05 determined statistical significance for the overall effect of the intervention.

### Ethical approval and consent to participate

The protocol for this meta-analysis is available in PROSPERO (CRD42022332185).

## Results

### Study selection process

**Figure 2:**
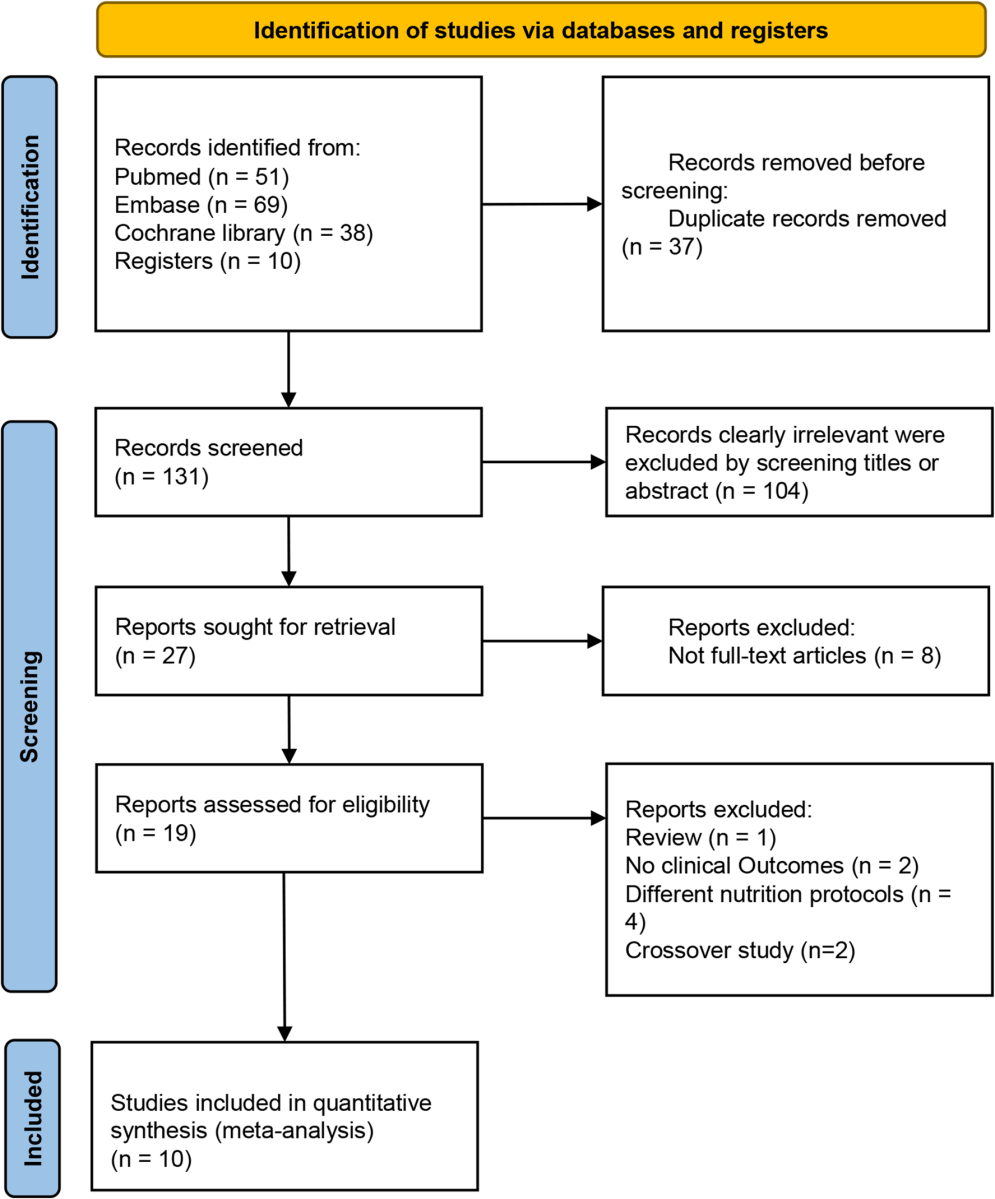
PRISMA flow chart on selection and inclusion of studies.

The search identified 158 potential trials. Ten additional studies were found during cross-referencing and from the authors’ own reference collections. After removing 37 duplicates, 131 manuscripts underwent title and abstract screening, and 27 trials underwent full-text screening. Details of the study selection process are shown in Fig. 2. Ten RCTs met the inclusion criteria of our review and underwent data extraction. Among these studies, three were conducted in the USA, two were conducted in France, one was conducted in Brazil, one was conducted in Switzerland, one was conducted in the UK, one was conducted in the USA and UK, and one study was conducted in the USA and Canada. Six of these studies were single-centre studies, and four were multicentre studies. The characteristics of trials and their participants are presented in Table 2 and additional file 1: Table S4, respectively^31–40^. In addition, we explained how to include studies that met AGI criteria in additional file 1: Table S5. A comparison of the formulas used in trials (Small peptide formulas vs. standard polymeric formulas) is shown in additional file: Table S6.

**Table 2:** Characteristics of the trials included in this review (n = 10).

Included trials	Population	Study design	Primary AGI/secondary AGI	Intervention/Sample size	Nutrition objectives	EN nutrition protocols	Primary outcomes
Brinson et al.^31^,	Medical and surgical ICU patients	Multicentre	Primary AGI and Secondary AGI	Peptide enteral formula (n = 7) vs standard enteral formula (n = 5)	Calorie: 35 kcal/kg/d	Continuous feeding	The incidence of diarrhea was lower in the Peptide enteral formula group
(United States)						Start time: NA	
					Protein: NR	Initial rate: 20 ml/h	
						Duration: ≥ 14 d	
Meredith et al.^32^, (United States)	Trauma patients in ICU	Single-centre	Primary AGI and Secondary AGI	Peptide-based formula (n = 9) vs intact-protein formula (n = 9)	Calorie: according to Harris-benedict equation	Continuous feeding	The incidence of diarrhea was lower in the Peptide-based formula group
						Start time: Within 24–48 h after ICU admission	
					Protein: NR	Initial rate: 25 ml/h	
						Duration: ≥ 7 d	
Mowatt-Larssen et al.^33^, (United States)	Critically ill patients with acute injury and albumin < 30 g/dL	Single-centre	Primary AGI and Secondary AGI	Peptide formula (n = 21) vs standard enteral formula(n = 20)	Calorie: 35 kcal/kg/d	Continuous feeding	Prevalence of diarrhea and elevated gastric residuals were similar between groups
						Start time: NR	
					Protein: NR	Initial rate: 25–50 ml/h	
						Duration: ≥ 5 d	
Heimburger et al.^34^, (United States and United Kingdom)	Adult critically ill patients in ICU	Multicentre	Primary AGI and Secondary AGI	Small-peptide formula (n = 26) vs whole-protein formula(n = 24)	NR	Continuous feeding?	Serum prealbumin and fibronectin increased more significantly in the small-peptide group
						Start time: NR	
						Initial rate: NA	
						Duration: ≥ 5 d	
Tiengou et al.^35^, (France)	ICU patients with acute pancreatitis requiring jejunal nutrition	Single-centre	Primary AGI	Semi-elemental formula (n = 15) vs polymeric formula(n = 15)	Calorie: 35 kcal/kg/d	Continuous feeding ≥ 18 h per day	There was no difference in nutrient tolerance between the two groups
						Start time: Within 24 h of jejunal tube placement	
					Protein: 1.5 g/kg/d	Initial rate: 20.8–27.8 ml/h	
						Duration: ≥ 7 d	
de Aguilar-Nascimento et al.^36^, (Brazil)	Elderly patients admitted to ICU due to acute ischemic stroke	Single-centre	Only secondary AGI	Hydrolysed whey protein formula (n = 10) vs standard formula(n = 15)	Calorie: 35 kcal/kg/d	Continuous feeding	Mortality was similar between groups
						Start time: ≤ 48 h after ICU admission	
					Protein: 1.2 g/kg/d	Initial rate: 20 ml/h	
						Duration: ≥ 5 d	
Jakob et al.^37^, (Switzerland)	Medical and surgical ICU patients expected ICU stay ≥ 5 days and EN ≥ 3 days	Single-centre	Primary AGI and Secondary AGI	Semi-elemental formula (n = 46) vs whole-protein formula (n = 44)	Calorie: 25 kcal/kg/d	Continuous feeding	Incidence of diarrhea was similar between groups
						Start time: ≤ 72 h after ICU admission	
					Protein: NR	Initial rate: NA	
						Duration: ≥ 3 d	
Rice et al.^38^, (United States and Canada)	Overweight/obese mechanically ventilated critically ill patients	Multicentre	Only secondary AGI	High-whey peptides formula (n = 50) vs standard polymeric protein formula (n = 52)	Calorie: NR	Continuous feeding	High-whey peptides formula group can reduce hyperglycemic events
						Start time: ≤ 48 h after ICU admission	
					Protein: 1.5 g/kg/d	Initial rate: NA	
						Duration: ≥ 5 d	
Carteron et al.^39^, (France)	Brain-injured critically ill patients expected to be ventilated > 48 h	Single-centre	Only secondary AGI	Semi-elemental formula (n = 100) vs polymeric formula (n = 95)	Calorie: 30 kcal/kg/d	Continuous feeding	Energy intake goal achievement rate was similar between groups
						Start time: ≤ 36 h after ICU admission	
					Protein: NR	Initial rate: 21 ml/h	
						Duration: NR	
de Brito-Ashurst et al.^40^, (United Kingdom)	Mechanically ventilated patients expected to require EN starting within 48 h	Multicentre	Only secondary AGI	The new peptide formula (n = 13) vs standard formula (n = 13)	Calorie: 25 kcal/kg/d	Continuous feeding	Gastrointestinal tolerance was similar between groups
						Start time: ≤ 48 h after ICU admission	
					Protein: NR	Initial rate: 20 mL/h Duration: ≥ 5 d	

AGI: acute gastrointestinal injury, ICU: intensive care unit, EN: enteral nutrition, NR: not reported.

### Primary outcome

**Figure 3:**
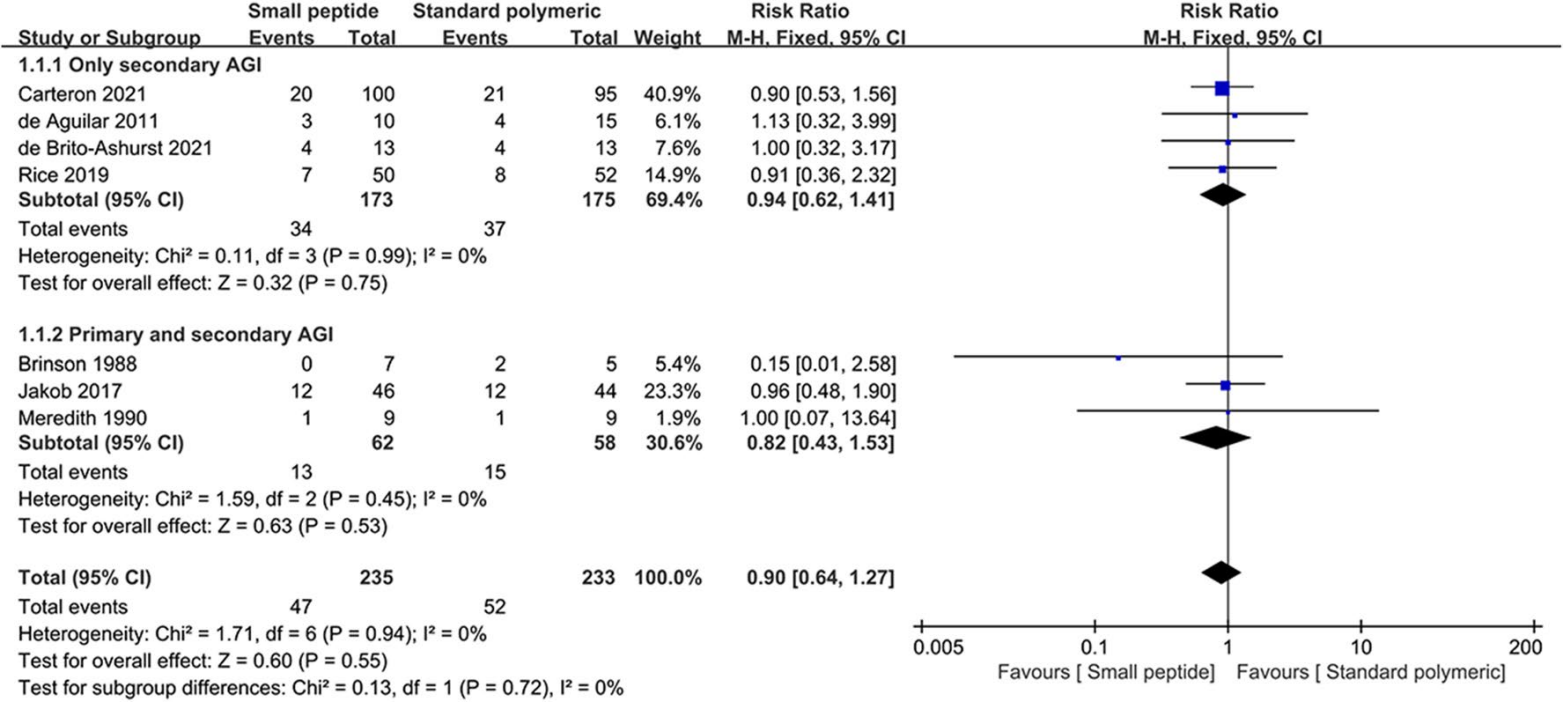
Forest plot for all-cause mortality.

Seven trials enrolling 468 patients reported all-cause mortality in small peptide formulas and standard polymeric formulas groups and were included in the meta-analysis. The pooled data showed that all-cause mortality was comparable between small peptide and standard polymeric formula groups (*RR* = 0.90; 95% *CI*, 0.64–1.27; *P* = 0.55; *Chi*^*2*^ = 1.71; *I*^*2*^ = 0%). In our subgroup analysis, there was no difference in the treatment effect between patients with only secondary AGI and those with primary and secondary AGI (subgroup difference test, *P* = 0.72) (Fig. 3).

### Secondary outcomes

#### Nutritional outcomes

##### Daily calorie intake

**Figure 4:**
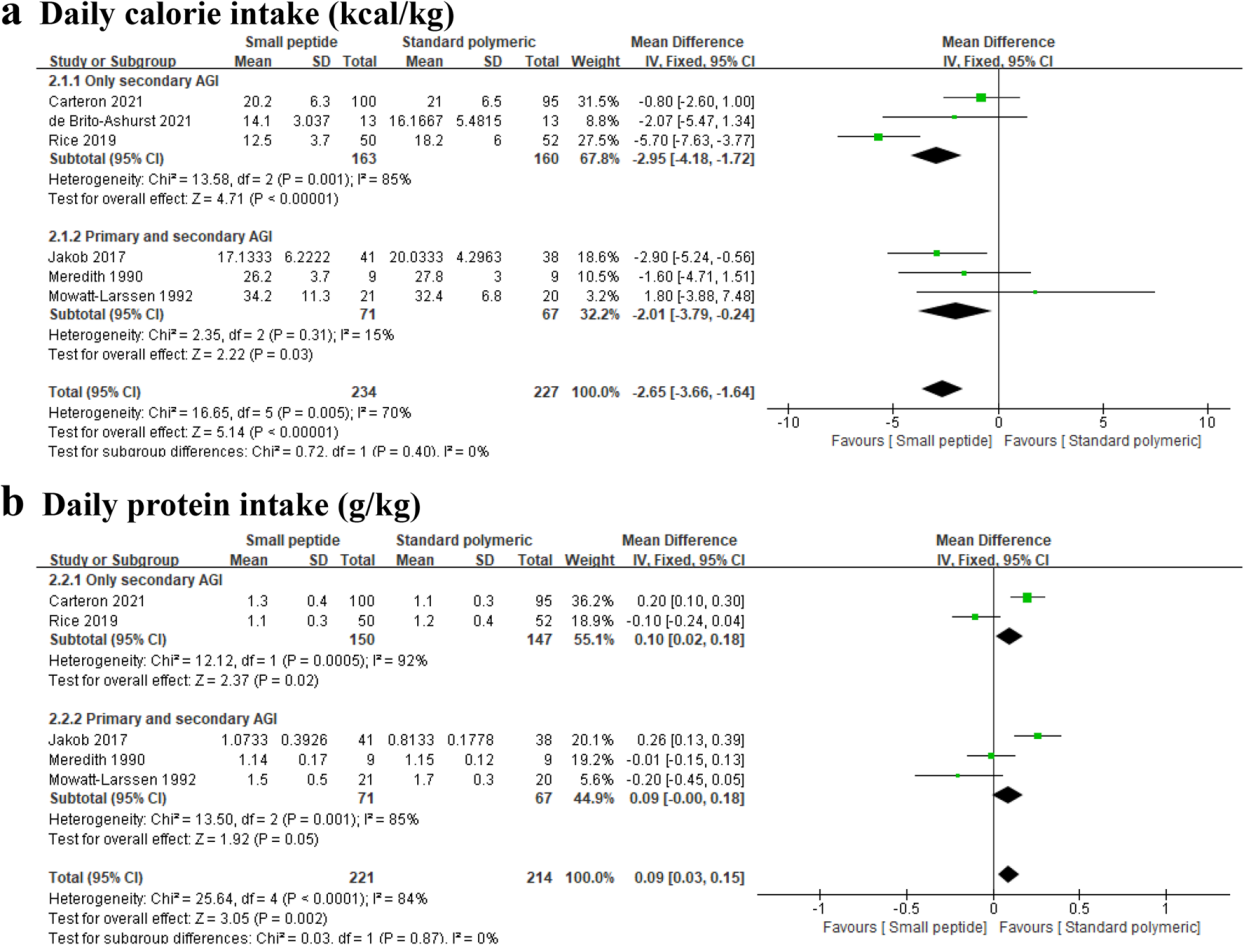
Forest plots for daily calorie intake (kcal/kg) (**a**) and daily protein intake (g/kg) (**b**).

Six trials enrolling 461 patients reported the daily calorie intake in small peptide formulas and standard polymeric formulas groups and were included in the meta-analysis. The pooled data showed that standard polymeric formulas could significantly increase the daily calorie intake compared with small peptide formulas (*MD* =  − 2.65; 95% *CI*, − 3.66 to − 1.64; *P* < 0.00001; *Chi*^*2*^ = 16.65; *I*^*2*^ = 70%). In our subgroup analysis, there was no difference in the daily calorie intake between patients with only secondary AGI and those with primary and secondary AGI (subgroup difference test, *P* = 0.40) (Fig. 4a).

##### Daily protein intake

Five trials enrolling 435 patients reported the daily calorie intake in small peptide formulas and standard polymeric formulas groups and were included in the meta-analysis. The pooled data showed a higher daily protein intake in the small peptide group compared with the standard polymeric formulas group (*MD* = 0.09; 95% *CI*, 0.03 to 0.15; *P* = 0.002; *Chi*^*2*^ = 25.64; *I*^*2*^ = 84%). In our subgroup analysis, there was no difference in the daily protein intake between patients with only secondary AGI and those with primary and secondary AGI (subgroup difference test, *P* = 0.87) (Fig. 4b).

##### Serum levels of albumin

Five trials reported the serum levels of albumin (albumin on the 5th day: three trials enrolling 261 patients; albumin on the 10th day: two trials enrolling 236 patients; albumin variation within 7 days: two trials enrolling 48 patients, respectively) in small peptide formulas and standard polymeric formulas groups, and were included in the meta-analysis. The pooled data showed that there was no statistically significant difference in the albumin on the 5th day and 10th day between small peptide formulas and standard polymeric formulas groups (*MD* =  − 0.03; 95% *CI*, − 0.12 to 0.07; *P* = 0.58; *Chi*^*2*^ = 17.12; *I*^*2*^ = 88% and *MD* =  − 0.04; 95% *CI*, − 0.14 to 0.07; *P* = 0.47; *Chi*^*2*^ = 0.72; *I*^*2*^ = 0%, respectively). However, the albumin variation within 7 days of the small peptide formulas group was higher than that of the standard polymeric formulas group (*MD* = 0.28; 95% *CI*, 0.10 to 0.45; *P* = 0.003; *Chi*^*2*^ = 0.47; *I*^*2*^ = 0%) (Additional file 1: Figure S1a).

##### Serum levels of prealbumin

Two trials enrolling 236 patients reported the serum levels of prealbumin in small peptide formulas and standard polymeric formulas groups and were included in the meta-analysis. The pooled data showed that the prealbumin on the 5th day was comparable between small peptide formulas and standard polymeric formulas groups (*MD* =  − 0.05; 95% *CI*, − 0.17 to 0.08; *P* = 0.47; *Chi*^*2*^ = 0.01; *I*^*2*^ = 0%). However, the prealbumin on the 10th day of the small peptide formulas group was higher than that of the standard polymeric formulas group (*MD* = 0.23; 95% *CI*, 0.06 to 0.40; *P* = 0.007; *Chi*^*2*^ = 2.73; *I*^*2*^ = 63%) (Additional file 1: Figure S1b).

##### Nitrogen balance

Three trials enrolling 56 patients reported the nitrogen balance in small peptide formulas and standard polymeric formulas groups and were included in the meta-analysis. The pooled data showed no significant difference between the small peptide formulas and standard polymeric formulas groups in nitrogen balance (*MD* =  − 0.15; 95% *CI*, − 1.21 to 0.90; *P* = 0.78; *Chi*^*2*^ = 1.53; *I*^*2*^ = 0%) (Additional file 1: Figure S1c).

#### Gastrointestinal adverse events

##### Diarrhea

Seven trials enrolling 431 patients reported the incidence of diarrhea in small peptide formulas and standard polymeric formulas groups and were included in the meta-analysis. The pooled data showed that the incidence of diarrhea was comparable between small peptide formulas and standard polymeric formulas groups (*RR* = 1.09; 95% *CI*, 0.83–1.41; *P* = 0.54; *Chi*^*2*^ = 10.78; *I*^*2*^ = 44%). In our subgroup analysis, there was no difference in the incidence of diarrhea between patients with only secondary AGI and those with primary and secondary AGI (subgroup difference test, *P* = 0.06) (Additional file 1: Figure S2a).

##### Gastric retention > 500 ml

Four trials enrolling 352 patients reported the incidence of gastric retention > 500 ml in small peptide formulas and standard polymeric formulas groups and were included in the meta-analysis. The pooled data showed that the incidence of gastric retention > 500 ml was comparable between small peptide formulas and standard polymeric formulas groups (*RR* = 1.49; 95% *CI*, 0.93–2.39; *P* = 0.09; *Chi*^*2*^ = 0.85; *I*^*2*^ = 0%). In our subgroup analysis, there was no difference in the incidence of gastric retention > 500 ml between patients with secondary AGI and those with primary or secondary AGI (subgroup difference test, *P* = 0.78) (Additional file 1: Figure S2b).

##### Vomiting

Two trials enrolling 116 patients reported the vomiting rate in small peptide formulas and standard polymeric formulas groups and were included in the meta-analysis. The pooled data showed no significant difference between small peptide formulas and standard polymeric formulas groups in vomiting rate (*RR* = 1.61; 95% *CI*, 0.65–3.98; *P* = 0.31; *Chi*^*2*^ = 0.07; *I*^*2*^ = 0%) (Additional file 1: Figure S2c).

#### Other clinical outcomes

##### ICU length of stay

Two trials enrolling 336 patients reported the ICU length of stay in small peptide formulas and standard polymeric formulas groups and were included in the meta-analysis. The pooled data showed that the small peptide formulas were associated with shorter ICU length of stay compared with standard polymeric formulas (*MD* =  − 2.23; 95% *CI*, − 3.56 to – 0.90; *P* = 0.001; *Chi*^*2*^ = 3.66; *I*^*2*^ = 18%) (Additional file 1: Figure S3a).

##### Hospital length of stay

Five trials enrolling 266 patients reported the hospital length of stay in small peptide formulas and standard polymeric formulas groups and were included in the meta-analysis. The pooled data showed that the small peptide formulas were associated with shorter ICU length of stay compared with standard polymeric formulas (*MD* =  − 1.85; 95% *CI*, − 2.54 to – 1.16; *P* < 0.0001; *Chi*^*2*^ = 50.60; *I*^*2*^ = 92%). In our subgroup analysis, there was a statistically significant difference in the hospital length of stay between patients with only secondary AGI and those with primary and secondary AGI (subgroup difference test, *P* < 0.00001). In the only secondary AGI subgroup, there was no statistically significant difference in the hospital length of stay between small peptide formulas and standard polymeric formulas groups (*MD* = 0.02; 95% *CI*, − 0.89 to 0.92; *P* = 0.97; *Chi*^*2*^ = 2.10; *I*^*2*^ = 52%). However, in the primary and secondary AGI subgroup, the small peptide formulas were associated with shorter hospital length of stay compared with standard polymeric formulas (*MD* =  − 4.37; 95% *CI*, − 5.43 to – 3.32; *P* < 0.0001; *Chi*^*2*^ = 10.12; *I*^*2*^ = 80%) (Additional file 1: Figure S3b).

##### Infections

Four trials enrolling 365 patients reported the incidence of infections in small peptide formulas and standard polymeric formulas groups and were included in the meta-analysis. The pooled data showed that the incidence of infections was comparable between small peptide formulas and standard polymeric formulas groups (*RR* = 0.97; 95% *CI*, 0.63–1.48; *P* = 0.87; *Chi*^*2*^ = 1.91; *I*^*2*^ = 0%) (Additional file 1: Figure S3c).

##### Mechanical ventilation duration

Two trials enrolling 285 patients reported the mechanical ventilation duration in small peptide formulas and standard polymeric formulas groups and were included in the meta-analysis. The pooled data showed that the mechanical ventilation duration was comparable between small peptide formulas and standard polymeric formulas groups (*MD* =  − 0.47; 95% *CI*, − 1.78 to 0.30; *P* = 0.16; *Chi*^*2*^ = 0.01;* I*^*2*^ = 0%) (Additional file 1: Figure S3d).

### Risk of bias and sensitivity analyses within outcomes

Funnel plots were used to assess publication bias for all outcomes (Additional file 1: Figure S4). According to the one-study-out method, the result of the sensitivity analysis of albumin variation within 7 days was the opposite after excluding the study by Tiengou et al.^35^, which might be influenced by the small number of included studies and the large difference in sample size between studies. Sensitivity analyses showed that the results of the primary outcome and other secondary outcomes were stable (Additional file 1: Figure S5).

## Discussion

This systematic review and meta-analysis of ten trials, including 589 patients, compared clinical outcomes of small peptide formulas with standard polymeric formulas in critically ill patients with AGI. We did not find significant differences in all-cause mortality between the small peptide formulas group and the standard polymer formulas group. On the other hand, while the daily calorie intake of the small peptide formulas group was lower than that of the standard polymeric formulas group, compared with the standard polymer formulas group, the small peptide formulas group had a higher daily protein intake, higher serum levels of albumin elevation within 7 days, higher serum levels of prealbumin on the 10th day, and shorter ICU and hospital length of stay. However, the two groups found no differences in nitrogen balance, the incidence of gastrointestinal adverse events, infection, and mechanical ventilation duration.

The findings of this review and the 7 trials that reported the primary clinical outcomes are consistent with other similar clinical studies, none of which found a difference in the impact of small peptide formulas and standard polymer formulas on mortality in critically ill patients with AGI^41–43^. David et al.^42^ found that all deaths in their study seem to be related to the patients’ underlying clinical condition but not feeding. Rational selection of EN formulas and optimization of EN feeding strategies may reduce the incidence of gastrointestinal adverse events, improve nutritional status, and increase muscle protein synthesis, but the effect on short-term mortality (< 90 days) is ignorable, and this primary outcome meets our expectations. However, due to the small sample size and heterogeneity among studies (such as differences in feeding regimen, characteristics of study subjects, and study design), the reliability of the results will be reduced, and a large sample size of RCTs is still needed to further verify the impact of nutrition formulas on mortality. Whether there is a long-term clinical outcome measure to evaluate the impact of nutritional formulas on critically ill patients with AGI needs to be further explored.

We did not find a significant difference in calorie density between the two formulas. However, this study found that standard polymer formulas provided higher daily calorie intake for critically ill patients with AGI, which is consistent with Rice et al.^38^. The possible reasons were as follows: first, the protein density of the standard polymer formulas was lower in these studies^37–39^, so the standard polymer formulas group required higher doses of enteral feeding to achieve the same protein goals. This resulted in higher daily calorie intake in the standard polymer formulas group. Secondly, the higher osmolality of small peptide formulas could promote osmotic diarrhea. Although the study by Carteron et al.^39^ reported no difference in the incidence of feeding intolerance between the small peptide formulas and standard polymer formulas groups, significantly more patients in the small peptide formulas group required dilution or reduction of the formulas due to severe diarrhea compared with the standard polymer formulas. These two factors may have resulted in a relative reduction in the amount of enteral feeding in the small peptide formulas group compared with the standard polymer formulas group, resulting in lower daily calorie intake. Although higher calorie intake may meet the nutritional needs of critically ill patients, several trials have demonstrated that meeting short-term caloric goals is of little or no significant clinical benefit^44–47^. A higher calorie intake in the acute phase of critical illness may increase the burden of mitochondrial oxidative metabolism, cause autophagy disorders, which can lead to persistent cell damage and dysfunction, and Higher mortality^48–50^.

Annika et al.^51^ showed that three or more gastrointestinal symptoms on the first day in the ICU were independently associated with a threefold increased risk of mortality. Hu et al.^19^ showed that the persistence of gastrointestinal adverse events during the first week of ICU stay is an independent determinant of mortality. Therefore, reducing the incidence of gastrointestinal adverse events is related to the clinical outcomes of critically ill patients with AGI. Carteron et al.^39^ reported more patients requiring clinical intervention (Requiring the addition of saline or loperamide) for diarrhea in the small peptide formulas group. The opposite argument had also been made; a RCT performed in multiple ICUs showed^42^ that pre-digested small peptide formulas reduced the incidence of gastrointestinal adverse events. However, in this study, despite the small peptide formula having higher osmolality, especially in the study by Meredith et al. (490 VS 310 mOsm/L)^32^, there were no differences in the incidence of diarrhea, gastric retention > 500 ml, and vomiting between the two groups, which was consistent with some studies^37,40^. High-quality RCTs are still needed to validate the difference between using the two formulas in the incidence of gastrointestinal adverse events.

Small peptide formulas can accelerate protein digestion and absorption from the gut, augment postprandial amino acid availability and have a tendency to increase the incorporation rate of dietary amino acids into skeletal muscle protein^52^. In addition, the intestinal protective effect of the small peptide formulas has been demonstrated in animal experiments^22–24^. As mentioned above, small peptide formulas may be more suitable for critically ill patients with AGI. Daily protein intake was higher in the small peptide group compared to the standard polymer group, which may be related to the additional whey protein in the formula. Protein reach goal is more critical than calorie requirements for critically ill patients with AGI, considering protein is the most important macronutrient for supporting immune function, healing wounds, and reducing muscle loss^53^. Studies have shown (Allingstrup et al., 2012) that a higher provision of protein and amino acids is associated with lower mortality compared to higher calories. This study also shows that the small peptide formulas group had a higher elevation in serum levels of albumin during 7 days and higher serum levels of prealbumin on the 10th day. This may be explained that the small peptide formulation can be absorbed without trypsin digestion and has a higher utilization rate^52^. In addition, small peptide formulation benefits in reduced ICU and hospital length of stay since adequate protein supplementation and absorption can lead to better nutritional status and higher muscle mass (respiratory muscle, skeletal muscle, etc.) in critically ill patients with AGI^54^. We found an interesting problem, the difference in the hospital length of stay was found in the primary and secondary AGI subgroup, but it was not found in the only secondary AGI subgroup. This outcome was associated with more severe intestinal damage in patients with primary AGI and thus may be more reliant on small peptide formulas. For serum albumin and prealbumin levels, only a few studies have been fully reported, so the results are volatile and further high-quality studies are needed to confirm the results. In the case of critically ill patients with AGI, it appears that standard polymer formulas may not fully exploit the advantages of physiological relevance, while small peptide formulas more suitable for critically ill patients with AGI.

Based on the findings of this study, it is evident that there is a considerable degree of heterogeneity in the meta-analysis, with I^2^ values exceeding 50% and even surpassing 75% for multiple secondary outcomes. Despite our efforts to minimize heterogeneity through subgroup analyses, significant heterogeneity persisted within subgroups, particularly for the three secondary outcomes: daily calorie intake, daily protein intake, and length of hospital stay. The observed high heterogeneity can be attributed to variations in formula composition across studies, differences in feeding management, variations in the characteristics of study subjects, and disparities in ICU types. These factors contribute to divergent outcomes among the studies analyzed, thereby undermining the robustness of the meta-analysis results. Additionally, there is a potential for publication bias, as certain studies reported positive outcomes despite having inadequate sample sizes to validate clinical findings. To address the issue of heterogeneity, sensitivity analysis was conducted. However, the results of this analysis did not yield significant findings for the majority of the secondary outcomes, suggesting that it did not effectively identify the studies with the highest levels of heterogeneity. In summary, the presence of high heterogeneity undermines the confidence in most of the secondary clinical outcomes of this study. Further research of superior quality is necessary to validate and reinforce the findings presented herein.

There is a degree of controversy about which EN formula should be used in critically ill patients with AGI. The results of some high-quality clinical studies were inconsistent, which will limit the rationalization of EN strategies. We conducted a meta-analysis of the data collected from these high-quality clinical trials to answer this controversial question in clinical practice. Therefore, this study is very necessary and meaningful.

There are several limitations in our meta-analysis. Firstly, the number of included trials was small, and most RCTs included a small number of patients. Further large-scale clinical trials should be conducted to confirm these results. Secondly, three trials did not describe the primary outcome of this study; we reached out to the corresponding author but did not receive a reply. Thirdly, due to the limitation of enrolled trials, our primary outcome of all-cause mortality included 28-day mortality, 90-day mortality, and hospital mortality. It is well known that these mortality rates are not interchangeable, and they depend on the mortality provided in each included trial. Fourthly, the data pertaining to secondary outcomes in certain reports exhibit a significant degree of heterogeneity, with only a limited number of studies providing descriptions for some of these secondary outcomes. As a result, the reliability of the outcomes may be compromised. Therefore, our results should be interpreted with caution. Fifthly, trials with different EN feeding strategies have been excluded from this study. However, there are still differences in feeding strategies among trials, which may result in a certain degree of bias.

## Conclusions

The choice of formula may not affect mortality in critically ill patients with AGI. Small peptide formulas were more conducive to increase daily protein intake, decrease intensive care unit and hospital length of stay. Further large-scale randomized controlled trials evaluating the effects of these two nutritional formulas on clinical and nutritional outcomes in critically ill patients with AGI are needed to confirm these results.

### Supplementary Information

Supplementary Information.

## Data Availability

All data generated or analyzed during this study are included in this published article.

## Abbreviations

AGI: Acute gastrointestinal injury
ICU: Intensive care unit
EN: Enteral nutrition
RCTs: Randomized controlled trials
RR: Relative risk
CI: Confidence interval
APACHE II: Acute Physiology and Chronic Health Evaluation II
