# Supplementary material for: Small peptide formulas versus standard polymeric formulas in critically ill patients with acute gastrointestinal injury: a systematic review and meta-analysis
Source: Sci Rep. 2023 Nov 22;13:20469. doi: 10.1038/s41598-023-47422-z (PMC10665341; doi:10.1038/s41598-023-47422-z)
Supplement: Supplementary file 1 — Supplementary Information. [file 41598_2023_47422_MOESM1_ESM.docx]

**Small peptide formulas vs. standard polymeric formulas in critically ill patients with acute gastrointestinal injury: [a systematic review and](https://www.pmop.cn/pubmed/35794612)meta-analysis.**

Youquan Wang^1^, Yanhua Li^1^, Hongxiang Li^1^, Yuting Li^1^, Xinyu Li^1^, Dong Zhang^1,^ *.

*^1^*Department of Critical Care Medicine, The First Hospital of Jilin University, Changchun, China

**Supplementary Information**

Supplementary material Table S1: PRISMA Checklist.

Supplementary material Table S2: AGI different grades.

Supplementary material Table S3: Search Strategy for Each Database.

Supplementary material Table S4: Characteristics of the Included Participants in each trial.

Supplementary material Table S5: Reasons why the included studies met AGI criteria.

Supplementary material Table S6: Small peptide formulas vs Standard polymeric formulas.

Supplementary material Figure S1: Forest plot for Serum levels of albumin, Serum levels of prealbumin and Nitrogen balance.

Supplementary material Figure S2: Forest plot for diarrhea, gastric residual > 500 ml and vomiting.

Supplementary material Figure S3. Forest plot for ICU and hospital length of stay, infections and mechanical ventilation duration.

Supplementary material Figure S4. Funnel plots for clinical and nutritional outcomes.

Supplementary material Figure S5. Sensitivity analyses clinical and nutritional outcomes.

**Supplementary material Table S1:** PRISMA Checklist.

| **Section/topic** | **#** | **Checklist item** | **Reported on page #** |
| --- | --- | --- | --- |
| **TITLE** | | |  |
| Title | 1 | Identify the report as a systematic review, meta-analysis, or both. | 1 |
| **ABSTRACT** | | |  |
| Structured summary | 2 | Provide a structured summary including, as applicable: background; objectives; data sources; study eligibility criteria, participants, and interventions; study appraisal and synthesis methods; results; limitations; conclusions and implications of key findings; systematic review registration number. | 2-3 |
| **INTRODUCTION** | | |  |
| Rationale | 3 | Describe the rationale for the review in the context of what is already known. | 4-5 |
| Objectives | 4 | Provide an explicit statement of questions being addressed with reference to participants, interventions, comparisons, outcomes, and study design (PICOS). | 6-7 |
| **METHODS** | | |  |
| Protocol and registration | 5 | Indicate if a review protocol exists, if and where it can be accessed (e.g., Web address), and, if available, provide registration information including registration number. | 6 |
| Eligibility criteria | 6 | Specify study characteristics (e.g., PICOS, length of follow-up) and report characteristics (e.g., years considered, language, publication status) used as criteria for eligibility, giving rationale. | 6-7 |
| Information sources | 7 | Describe all information sources (e.g., databases with dates of coverage, contact with study authors to identify additional studies) in the search and date last searched. | 8 |
| Search | 8 | Present full electronic search strategy for at least one database, including any limits used, such that it could be repeated. | 8 |
| Study selection | 9 | State the process for selecting studies (i.e., screening, eligibility, included in systematic review, and, if applicable, included in the meta-analysis). | 8 |
| Data collection process | 10 | Describe method of data extraction from reports (e.g., piloted forms, independently, in duplicate) and any processes for obtaining and confirming data from investigators. | 8-9 |
| Data items | 11 | List and define all variables for which data were sought (e.g., PICOS, funding sources) and any assumptions and simplifications made. | 8-9 |
| Risk of bias in individual studies | 12 | Describe methods used for assessing risk of bias of individual studies (including specification of whether this was done at the study or outcome level), and how this information is to be used in any data synthesis. | 9 |
| Summary measures | 13 | State the principal summary measures (e.g., risk ratio, difference in means). | 9-10 |
| Synthesis of results | 14 | Describe the methods of handling data and combining results of studies, if done, including measures of consistency (e.g., I^2^) for each meta-analysis. | 10 |
| Risk of bias across studies | 15 | Specify any assessment of risk of bias that may affect the cumulative evidence (e.g., publication bias, selective reporting within studies). | 9 |
| Additional analyses | 16 | Describe methods of additional analyses (e.g., sensitivity or subgroup analyses, meta-regression), if done, indicating which were pre-specified. | 10 |
| **RESULTS** | | |  |
| Study selection | 17 | Give numbers of studies screened, assessed for eligibility, and included in the review, with reasons for exclusions at each stage, ideally with a flow diagram. | 10 |
| Study characteristics | 18 | For each study, present characteristics for which data were extracted (e.g., study size, PICOS, follow-up period) and provide the citations. | 11-15 |
| Risk of bias within studies | 19 | Present data on risk of bias of each study and, if available, any outcome level assessment (see item 12). | 9 |
| Results of individual studies | 20 | For all outcomes considered (benefits or harms), present, for each study: (a) simple summary data for each intervention group (b) effect estimates and confidence intervals, ideally with a forest plot. | 16-22 |
| Synthesis of results | 21 | Present results of each meta-analysis done, including confidence intervals and measures of consistency. | 16-22 |
| Risk of bias across studies | 22 | Present results of any assessment of risk of bias across studies (see Item 15). | 22-23 |
| Additional analysis | 23 | Give results of additional analyses, if done (e.g., sensitivity or subgroup analyses, meta-regression [see Item 16]). | 22-23 |
| **DISCUSSION** | | |  |
| Summary of evidence | 24 | Summarize the main findings including the strength of evidence for each main outcome; consider their relevance to key groups (e.g., healthcare providers, users, and policy makers). | 23-27 |
| Limitations | 25 | Discuss limitations at study and outcome level (e.g., risk of bias), and at review-level (e.g., incomplete retrieval of identified research, reporting bias). | 27 |
| Conclusions | 26 | Provide a general interpretation of the results in the context of other evidence, and implications for future research. | 27-28 |
| **FUNDING** | | |  |
| Funding | 27 | Describe sources of funding for the systematic review and other support (e.g., supply of data); role of funders for the systematic review. | 30 |

*From:* Moher D, Liberati A, Tetzlaff J, Altman DG, The PRISMA Group (2009). Preferred Reporting Items for Systematic Reviews and Meta-Analyses: The PRISMA Statement. PLoS Med 6(6): e1000097. doi:10.1371/journal.pmed1000097

**Table S2.** **AGI different grades(1)**

| AGI grades | Diagnostic criteria |
| --- | --- |
| AGI grade I | the function of the GI tract is partially impaired, expressed as GI symptoms related to a known cause and perceived as transient. |
| AGI grade II | the GI tract cannot perform digestion and absorption adequately to satisfy the nutrient and fluid requirements of the body. There are no changes in the patient’s general condition related to GI problems. |
| AGI grade III | loss of GI function, where restoration of GI function is not achieved despite interventions and the general condition is not improving. |
| AGI grade IV | AGI has progressed to become directly and immediately life-threatening, worsening MODS and shock. |

Abbreviation: AGI, acute gastrointestinal injury; GI, gastrointestinal; MODS, multiple organ dysfunction syndrome

**Table S3.** **Search Strategy for Each Database**

| Database | Search strategy |
| --- | --- |
| Pubmed | #1 ((((((((((((((((((((("Peptides"[Mesh]) OR (Peptide[Title/Abstract])) OR (Polypeptides[Title/Abstract])) OR (Polypeptide[Title/Abstract])) OR ("Dietary Proteins"[Mesh])) OR (Proteins, Dietary[Title/Abstract])) OR (Dietary Protein[Title/Abstract])) OR (Protein, Dietary[Title/Abstract])) OR ("Oligopeptides"[Mesh])) OR (Oligopeptide[Title/Abstract])) OR ("Whey Proteins"[Mesh])) OR (Protein, Whey[Title/Abstract])) OR (Proteins, Whey[Title/Abstract])) OR (Whey Protein[Title/Abstract])) OR ("Protein Hydrolysates"[Mesh])) OR (Hydrolysates, Protein[Title/Abstract])) OR (Protein Hydrolysate[Title/Abstract])) OR (Hydrolysate, Protein[Title/Abstract])) OR (semi-elemental[Title/Abstract])) OR (semi elemental[Title/Abstract])) OR (Peptamen[Title/Abstract])) |
|  | #2 ((((((((whole protein[Title/Abstract]) OR (polymeric[Title/Abstract])) OR (standard[Title/Abstract])) OR (intact protein[Title/Abstract])) OR (whole-protein[Title/Abstract])) OR (casein protein[Title/Abstract])) OR (intact-protein[Title/Abstract])) OR (casein-protein[Title/Abstract])) |
|  | #3 (((((((((((((((((((((("Food, Formulated"[Mesh]) OR (Foods, Formulated[Title/Abstract])) OR (Formulated Food[Title/Abstract])) OR (Formulated Foods[Title/Abstract])) OR (Synthetic Diet[Title/Abstract])) OR (Diets, Synthetic[Title/Abstract])) OR (Diet, Synthetic[Title/Abstract])) OR (Diet, Formula[Title/Abstract])) OR (Diets, Formula[Title/Abstract])) OR (Dietary Formulations[Title/Abstract])) OR (Dietary Formulation[Title/Abstract])) OR (Formulation, Dietary[Title/Abstract])) OR (Formulations, Dietary[Title/Abstract])) OR (Diet, Chemically Defined[Title/Abstract])) OR (Chemically Defined Diet[Title/Abstract])) OR (Chemically Defined Diets[Title/Abstract])) OR (Diets, Chemically Defined[Title/Abstract])) OR (Diet, Elemental[Title/Abstract])) OR (Diets, Elemental[Title/Abstract])) OR (Elemental Diet[Title/Abstract])) OR (Elemental Diets[Title/Abstract])) OR (diet[Title/Abstract])) |
|  | #4 (((((((((("Critical Care"[Mesh]) OR (Care, Critical[Title/Abstract])) OR (Intensive Care[Title/Abstract])) OR (Care, Intensive[Title/Abstract])) OR (Surgical Intensive Care[Title/Abstract])) OR (Care, Surgical Intensive[Title/Abstract])) OR (Intensive Care, Surgical[Title/Abstract]))) OR ((((("Critical Illness"[Mesh]) OR (Critical Illnesses[Title/Abstract])) OR (Illness, Critical[Title/Abstract])) OR (Illnesses, Critical[Title/Abstract])) OR (Critically Ill[Title/Abstract]))) OR (((("Intensive Care Units"[Mesh]) OR (Intensive Care Unit[Title/Abstract])) OR (Unit, Intensive Care[Title/Abstract])) OR (ICU Intensive Care Units[Title/Abstract]))) |
|  | #5 #1 AND #2 AND #3 AND #4 |
| Embase | #1 'peptide'/exp  #2 'nucleotide peptide':ab,ti OR 'ovoid peptide':ab,ti OR 'peptide chain':ab,ti OR 'peptide fraction':ab,ti OR 'peptide group':ab,ti OR 'peptides':ab,ti OR 'peptidyl group':ab,ti OR 'permethylated peptide':ab,ti OR 'short chain peptide':ab,ti  #3 'whey protein'/exp  #4 ‘beneprotein’:ab,ti OR 'milk whey protein':ab,ti OR 'resource whey protein':ab,ti OR vitapro:ab,ti OR 'whey proteins':ab,ti  #5 'elemental diet'/exp  #6 'semi elemental':ab,ti OR peptamen:ab,ti  #7 'whole protein':ab,ti OR polymeric:ab,ti OR standard:ab,ti OR 'intact protein':ab,ti OR 'casein protein':ab,ti  #8 'nutrition supplement'/exp  #9 'enteric feeding'/exp  #10 'enteral nutrition product':ab,ti OR 'nutrition mixture':ab,ti OR 'nutrition support product':ab,ti OR 'nutritional mixture':ab,ti OR 'nutritional supplement':ab,ti OR 'enteral feeding':ab,ti OR 'enteral nutrition':ab,ti OR 'enteric nutrition':ab,ti OR 'feeding, enteric':ab,ti OR 'feeding, intragastric':ab,ti OR 'intestinal feeding':ab,ti OR 'intragastric feeding':ab,ti OR 'intraintestinal feeding':ab,ti OR 'tube feeding':ab,ti  #11 'diet'/exp  #12 'diet influence':ab,ti OR 'diet regimen':ab,ti OR 'diet surveys':ab,ti OR 'dietary effect':ab,ti OR 'dietary influence':ab,ti OR 'dietary survey':ab,ti OR 'dietary surveys':ab,ti OR dieting:ab,ti  #13 'intensive care'/exp  #14 'care, intensive':ab,ti OR 'critical care':ab,ti OR 'intensive therapy':ab,ti OR 'therapy, intensive':ab,ti  #15 'intensive care unit'/exp  #16 'close attention unit':ab,ti OR ('combined medical':ab,ti AND 'surgical icu':ab,ti) OR ('combined surgical':ab,ti AND 'medical icu':ab,ti) OR 'critical care unit':ab,ti OR 'general icu':ab,ti OR gicu:ab,ti OR gicus:ab,ti OR icus:ab,ti OR 'intensive care department':ab,ti OR 'intensive care units':ab,ti OR 'intensive therapy unit':ab,ti OR 'intensive treatment unit':ab,ti OR 'medical-surgery icu':ab,ti OR 'medical/surgical icu':ab,ti OR 'medical/surgical icus':ab,ti OR 'medico-surgical icu':ab,ti OR ('mixed medical':ab,ti AND 'surgical icu':ab,ti) OR ('mixed surgical':ab,ti AND 'medical icu':ab,ti) OR 'respiratory care unit':ab,ti OR 'respiratory care units':ab,ti OR 'special care unit':ab,ti OR 'surgery/medical icu':ab,ti OR 'surgical-medical icus':ab,ti OR 'surgical/medical icu':ab,ti OR 'unit, intensive care':ab,ti  #17 #1 OR #2 OR #3 OR #4 OR #5 OR #6  #18 #7 AND #17  #19 #8 OR #9 OR #10 OR #11 OR #12  #20 #13 OR #14 OR #15 OR #16  #21 #18 AND #19 AND #20  #22 #18 AND #19 AND #20 AND [randomized controlled trial]/lim |
| Cochrane  library | #1 MeSH descriptor: [Peptides] explode all trees  #2 MeSH descriptor: [Dietary Proteins] explode all trees  #3 MeSH descriptor: [Oligopeptides] explode all trees  #4 MeSH descriptor: [Whey Proteins] explode all trees  #5 (“Peptides” OR “Peptide” OR “Polypeptides” OR “Polypeptide” OR “Dietary Proteins” OR “Proteins, Dietary” OR “Dietary Protein” OR “Protein, Dietary” OR “Oligopeptides” OR “Whey Proteins” OR “Protein, Whey” OR “Proteins, Whey” OR “Whey Protein” OR “Protein Hydrolysates” OR “Hydrolysates, Protein” OR “Protein Hydrolysate” OR “Hydrolysate, Protein” OR “semi-elemental” OR “semi elemental” OR “Peptamen”):ti,ab,kw  #6 #1 or #2 or #3 or #4 or #5  #7 (“whole protein” OR “polymeric” OR “standard” OR “intact protein” OR “whole-protein” OR “casein protein” OR “casein-protein” OR “intact-protein”):ti,ab,kw  #8 #6 and #7  #9 MeSH descriptor: [Food, Formulated] explode all trees  #10 (“Food, Formulated” OR “Foods, Formulated” OR “Formulated Food” OR “Formulated Foods” OR “Synthetic Diet” OR “Diets, Synthetic” OR “Diet, Synthetic” OR “Diet, Formula” OR “Diets, Formula” OR “Dietary Formulations” OR “Dietary Formulation” OR “Formulation, Dietary” OR “Formulations, Dietary” OR “Diet, Chemically Defined” OR “Chemically Defined Diet” OR “Chemically Defined Diets” OR “Diets, Chemically Defined” OR “Diet, Elemental” OR “Diets, Elemental” OR “Elemental Diet” OR “Elemental Diets” OR “diet”)):ti,ab,kw  #11 #9 or #10  #12 #8 and #11  #13 MeSH descriptor: [Critical Care] explode all trees  #14 MeSH descriptor: [Critical Illness] explode all trees  #15 MeSH descriptor: [Intensive Care Units] explode all trees  #16 (“Critical Care” OR “Care, Critical” OR “Intensive Care” OR “Care, Intensive” OR “Surgical Intensive Care” OR “Care, Surgical Intensive” OR “Intensive Care, Surgical” OR “Critical Illness” OR “Critical Illnesses” OR “Illness, Critical” OR “Illnesses, Critical” OR “Critically Ill” OR “Intensive Care Units” OR “Intensive Care Unit” OR “Unit, Intensive Care” OR “ICU Intensive Care Units”):ti,ab,kw  #17 #13 or #14 or #15 or #16  #18 #12 and #17 |

**Table S4.** **Characteristics of the Included Participants in each trial**

| Included trials | Small peptide formulas | | | |  | Standard polymeric formulas | | | |
| --- | --- | --- | --- | --- | --- | --- | --- | --- | --- |
|  | Age (years) | Male^a^ | Albumin (g/dL) | APACHE II score |  | Age (years) | Male^a^ | Albumin (g/dL) | APACHE II score |
| Brinson et al(2) | 54 [38–70] ^c^ | 3 (43) | 2.2 ± 0.3 ^b^ | NR |  | 42 [18–66] ^c^ | 1 (20) | 2.0 ± 0.4 ^b^ | NR |
| Meredith et al(3) | 51.6 ± 7.8 ^b^ | NR | 2.7 ± 0.1 ^b^ | NR |  | 46.1 ± 6.4 ^b^ | NR | 2.5 ± 0.1 ^b^ | NR |
| Mowatt-Larssen et al(4) | 38.4 ± 19.8 ^b^ | 15 (71) | 2.5 ± 0.3 ^b^ | 9.5 ± 4.6 ^b^ |  | 41.1 ± 16.8 ^b^ | 15 (75) | 2.5 ± 0.4 ^b^ | 8.7 ± 3.6 ^b^ |
| Heimburger et al(5) | 59 ± 16 ^b^ | 13 (50) | 2.7 ± 0.5 ^b^ | NR |  | 45 ± 19 ^b^ | 16 (67) | 2.8 ± 0.7 ^b^ | NR |
| Tiengou et al(6) | 44 ± 3 ^b^ | 13 (87) | 3.9 ± 0.2 ^b^ | NR |  | 48 ± 4 ^b^ | 11 (73) | 3.6 ± 0.2 ^b^ | NR |
| de Aguilar-Nascimento et al(7) | 74 [67–88] ^c^ | 6 (60) | 3.1 ± 0.2 ^b^ | 16.5 [8–26] ^c^ |  | 75 [66–90] ^c^ | 3 (20) | 3.4 ± 0.1 ^b^ | 18 [8–28] ^c^ |
| Jakob et al(8) | 65 [53–75] ^c^ | 33 (72) | 2.1 [1.7–2.5] ^c^ | 28.5 [22.3–32.8] ^c^ |  | 62 [49–71] ^c^ | 28 (64) | 2.1 [1.8–2.4] ^c^ | 27.5 [22.0–33.3] ^c^ |
| Rice et al(9) | 63.3 ± 11.9 ^b^ | 24 (45) | NR | 24.8 ± 8.8 ^b^ |  | 61.0 ± 14.6 ^b^ | 30 (58) | NR | 25.9 ± 9.2 ^b^ |
| Carteron et al(10) | 57 [44–65] ^c^ | 67 (67) | 2.7 ± 0.4 ^b^ | NR |  | 55 [40–65] ^c^ | 53 (56) | 2.7 ± 0.4 ^b^ | NR |
| de Brito-Ashurst et al(11) | 57 ± 19 ^b^ | 8 (61) | NR | 26 [18−28] ^c^ |  | 55 ± 17 ^b^ | 6 (46) | NR | 21 [16−24] ^c^ |

^a^ Data are number of patients (percentage); ^b^ Mean ± SD; ^c^ Median [interquartile range].

Abbreviation: NR, not reported; APACHE, acute physiology and chronic health evaluation

**References**

1. Brinson, R.R. and B.E. Kolts, *Diarrhea associated with severe hypoalbuminemia: a comparison of a peptide-based chemically defined diet and standard enteral alimentation.* Crit Care Med, 1988. **16**(2): p. 130-6.

2. Meredith, J.W., J.A. Ditesheim, and G.P. Zaloga, *Visceral protein levels in trauma patients are greater with peptide diet than with intact protein diet.* J Trauma, 1990. **30**(7): p. 825-8; discussion 828-9.

3. Mowatt-Larssen, C.A., et al., *Comparison of tolerance and nutritional outcome between a peptide and a standard enteral formula in critically ill, hypoalbuminemic patients.* JPEN J Parenter Enteral Nutr, 1992. **16**(1): p. 20-4.

4. Heimburger, D.C., et al., *Effects of small-peptide and whole-protein enteral feedings on serum proteins and diarrhea in critically ill patients: a randomized trial.* JPEN J Parenter Enteral Nutr, 1997. **21**(3): p. 162-7.

5. Tiengou, L.E., et al., *Semi-elemental formula or polymeric formula: Is there a better choice for enteral nutrition in acute pancreatitis? Randomized comparative study.* Journal of Parenteral and Enteral Nutrition, 2006. **30**(1): p. 1-5.

6. de Aguilar-Nascimento, J.E., B.R. Prado Silveira, and D.B. Dock-Nascimento, *Early enteral nutrition with whey protein or casein in elderly patients with acute ischemic stroke: a double-blind randomized trial.* Nutrition, 2011. **27**(4): p. 440-4.

7. Jakob, S.M., et al., *A randomized controlled pilot study to evaluate the effect of an enteral formulation designed to improve gastrointestinal tolerance in the critically ill patient-the SPIRIT trial.* Critical Care, 2017. **21**(1).

8. Rice, T.W., et al., *Dietary Management of Blood Glucose in Medical Critically Ill Overweight and Obese Patients: An Open-Label Randomized Trial.* JPEN J Parenter Enteral Nutr, 2019. **43**(4): p. 471-480.

9. Carteron, L., et al., *Semi-elemental versus polymeric formula for enteral nutrition in brain-injured critically ill patients: a randomized trial.* Crit Care, 2021. **25**(1): p. 31.

10. de Brito-Ashurst, I., et al., *Gastrointestinal tolerance and protein absorption markers with a new peptide enteral formula compared to a standard intact protein enteral formula in critically ill patients.* Nutrients, 2021. **13**(7).

**Table S5.** **Reasons why the included studies met AGI criteria**

| Included trials | Reasons |
| --- | --- |
| Brinson et al(2) | Patients included in the study had multiple organ failure and were at high risk for GI adverse events |
| Meredith et al(3) | Patients included in the study had blunt or penetrating injuries to the abdomen and other organs and were at risk for feeding intolerance |
| Mowatt-Larssen et al(4) | Consecutive acutely injured, critically ill patients with  serum albumin concentrations less than 3.0 g/dL. And there are many feeding intolerances such as gastric retention and diarrhea |
| Heimburger et al(5) | Critically ill patients included in the study were at risk of sepsis and diarrhea |
| Tiengou et al(6) | All patients included in the study were patients with acute pancreatitis who had pancreatic damage and met the diagnostic criteria for primary AGI |
| de Aguilar-Nascimento et al(7) | All patients included in the study were elderly patients admitted to the ICU due to acute ischemic stroke who were at risk for feeding intolerance during the acute phase of the disease |
| Jakob et al(8) | This study included patients in the medical and surgical ICUs in the acute phase of the disease, at risk for feeding intolerance, and with a higher frequency of diarrhea, gastric retention and interruption of EN due to diarrhea |
| Rice et al(9) | All patients included in the study were mechanically ventilated, critically ill, overweight, and obese (body mass index 26–45) and required EN for 5 days or longer. They were at risk of developing GI dysfunction or failure |
| Carteron et al(10) | Included critically ill patients with brain injury with an initial GCS ≤ 8 and expected duration of mechanical ventilation > 48 h, they have organ failure and are at risk of developing GI dysfunction |
| de Brito-Ashurst et al(11) | All mechanically ventilated patients included in this study, they had a high incidence of GI adverse events（diarrhea, constipation, vomiting, and incidence of high gastric retention) in the first 5 days |

Abbreviation: AGI, acute gastrointestinal injury; GI, gastrointestinal; GSC, Glasgow Coma Score; ICU, intensive care unit; EN, enteral nutrition.

**Table S6.** **Small peptide formulas vs Standard polymeric formulas**

| Included trials | Small peptide formulas | | | |  | | Standard polymeric formulas | | | | |  |
| --- | --- | --- | --- | --- | --- | --- | --- | --- | --- | --- | --- | --- |
|  | Calories (kcal) ^a^ | Proteins (g) ^a^ | Osmolality（mOsm/L） | Carbohydrate(g) ^a^ | |  | | Calories（kcal）^a^ | Proteins (g) ^a^ | Osmolality（mOsm/L） | Carbohydrate(g) ^a^ | |
| Brinson et al(2) | 100 | 16.7% ^b^ | NR | NR | |  | | 100 | 16.7% ^b^ | NR | NR | |
| Meredith et al(3) | 133 | 5.8 | 490 | 15.8 | |  | | 106 | 4.44 | 310 | 14.1 | |
| Mowatt-Larssen et al(4), | NR | NR | NR | NR | |  | | NR | NR | NR | NR | |
| Heimburger et al(5), | 100 | 4.0 | 270-380 | NR | |  | | 100 | 4.0 | 300-350 | NR | |
| Tiengou et al(6) | 100 | 4.0 | NR | NR | |  | | 100 | 3.8 | NR | NR | |
| de Aguilar-Nascimento et al(7) | 150 | 6.7 | NR | 18.4 | |  | | 153 | 6.7 | NR | 18.4 | |
| Jakob et al(8) | 150 | 9.4 | 380 | 13.5 | |  | | 157 | 6.1 | 387 | 19.3 | |
| Rice et al(9) | 100 | 37 % ^c^ | 345 | 29 % ^c^ | |  | | 100 | 25% ^c^ | 300 | 45 % ^b^ | |
| Carteron et al(10) | 150 | 9.4 | 380 | 14.0 | |  | | 150 | 7.5 | 310 | 17.0 | |
| de Brito-Ashurst et al(11) | 150 | 7.5 | NR | 19.0 | |  | | 150 | 7.5 | NR | 16.9 | |

^a^: Per 100ml formulas; ^b^: 16.7% of calories as protein ^c^: Percentage of the formulas;

Abbreviation: NR, not reported.

**References**

1. Reintam Blaser A, Malbrain ML, Starkopf J, Fruhwald S, Jakob SM, De Waele J, et al. Gastrointestinal function in intensive care patients: terminology, definitions and management. Recommendations of the ESICM Working Group on Abdominal Problems. Intensive Care Med. 2012;38(3):384-94.

2. Brinson RR, Kolts BE. Diarrhea associated with severe hypoalbuminemia: a comparison of a peptide-based chemically defined diet and standard enteral alimentation. Crit Care Med. 1988;16(2):130-6.

3. Meredith JW, Ditesheim JA, Zaloga GP. Visceral protein levels in trauma patients are greater with peptide diet than with intact protein diet. J Trauma. 1990;30(7):825-8; discussion 8-9.

4. Mowatt-Larssen CA, Brown RO, Wojtysiak SL, Kudsk KA. Comparison of tolerance and nutritional outcome between a peptide and a standard enteral formula in critically ill, hypoalbuminemic patients. JPEN J Parenter Enteral Nutr. 1992;16(1):20-4.

5. Heimburger DC, Geels VJ, Bilbrey J, Redden DT, Keeney C. Effects of small-peptide and whole-protein enteral feedings on serum proteins and diarrhea in critically ill patients: a randomized trial. JPEN J Parenter Enteral Nutr. 1997;21(3):162-7.

6. Tiengou LE, Gloro R, Pouzoulet J, Bouhier K, Read MH, Arnaud-Battandier F, et al. Semi-elemental formula or polymeric formula: Is there a better choice for enteral nutrition in acute pancreatitis? Randomized comparative study. Journal of Parenteral and Enteral Nutrition. 2006;30(1):1-5.

7. de Aguilar-Nascimento JE, Prado Silveira BR, Dock-Nascimento DB. Early enteral nutrition with whey protein or casein in elderly patients with acute ischemic stroke: a double-blind randomized trial. Nutrition. 2011;27(4):440-4.

8. Jakob SM, Bütikofer L, Berger D, Coslovsky M, Takala J. A randomized controlled pilot study to evaluate the effect of an enteral formulation designed to improve gastrointestinal tolerance in the critically ill patient-the SPIRIT trial. Critical Care. 2017;21(1).

9. Rice TW, Files DC, Morris PE, Bernard AC, Ziegler TR, Drover JW, et al. Dietary Management of Blood Glucose in Medical Critically Ill Overweight and Obese Patients: An Open-Label Randomized Trial. JPEN J Parenter Enteral Nutr. 2019;43(4):471-80.

10. Carteron L, Samain E, Winiszewski H, Blasco G, Balon AS, Gilli C, et al. Semi-elemental versus polymeric formula for enteral nutrition in brain-injured critically ill patients: a randomized trial. Crit Care. 2021;25(1):31.

11. de Brito-Ashurst I, Klebach M, Tsompanaki E, Kaul S, van Horssen P, Hofman Z. Gastrointestinal tolerance and protein absorption markers with a new peptide enteral formula compared to a standard intact protein enteral formula in critically ill patients. Nutrients. 2021;13(7).


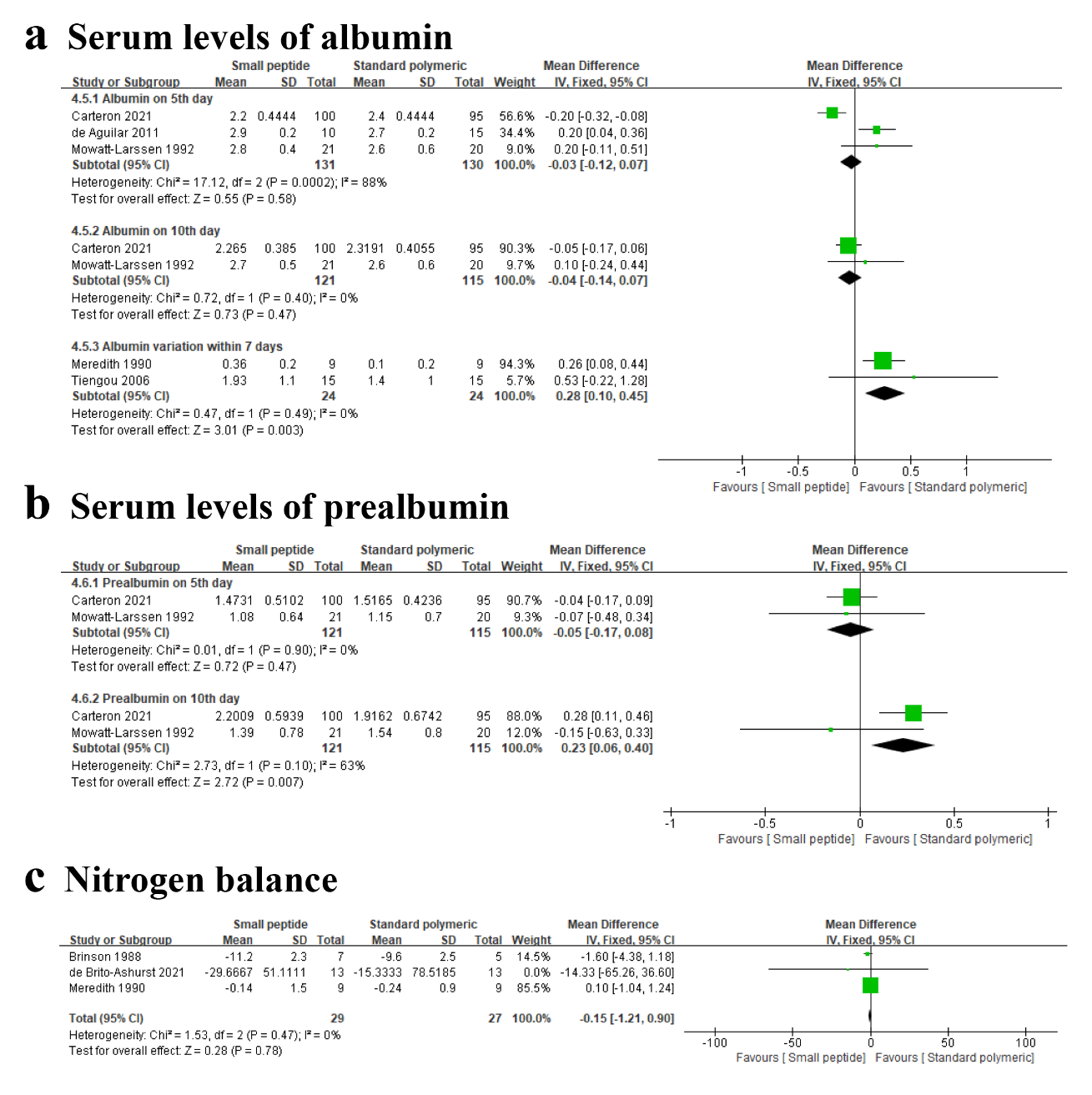


Figure S1. Forest plot for Serum levels of albumin (a), Serum levels of prealbumin (b) and Nitrogen balance (c).


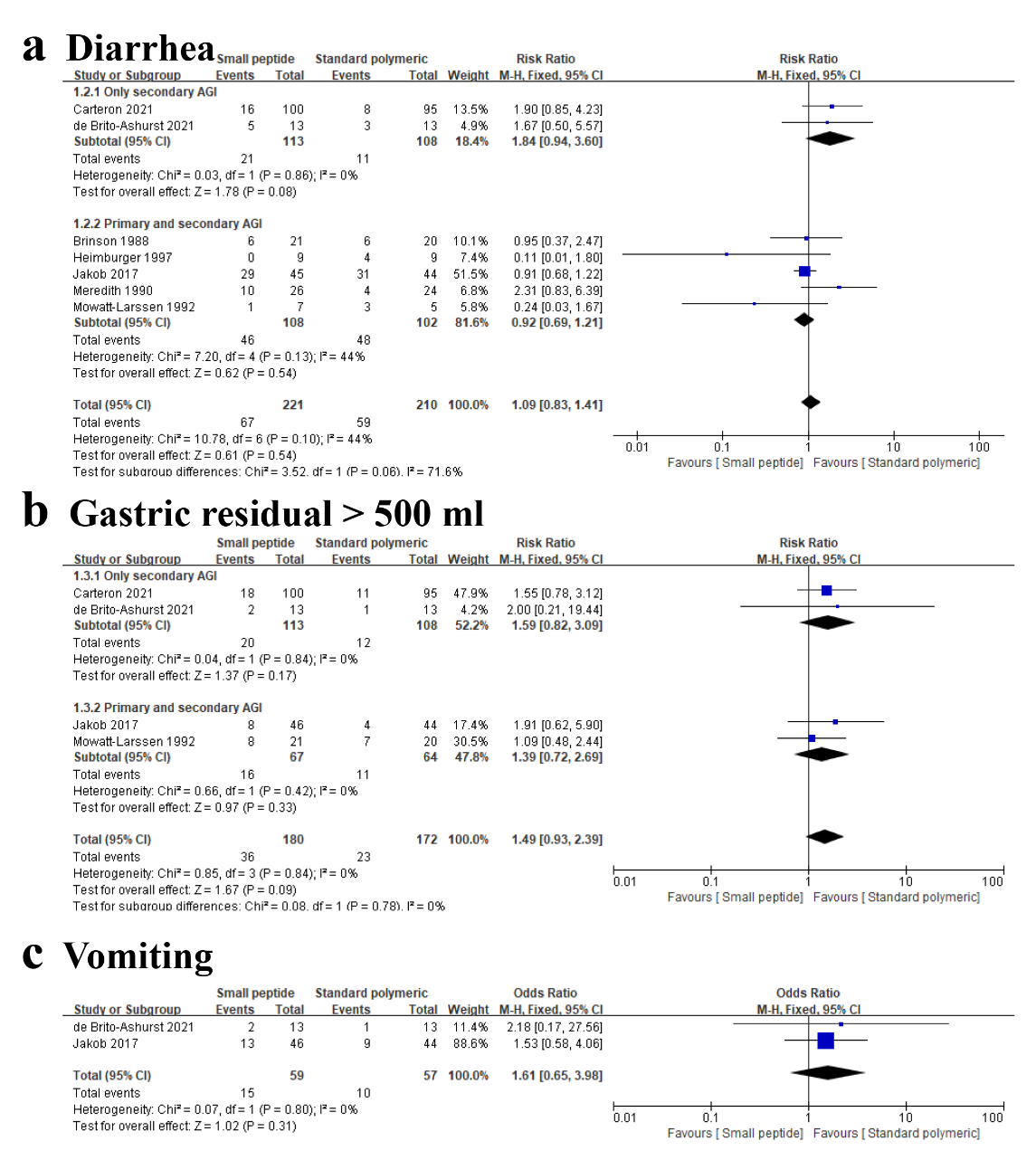


Figure S2. Forest plot for diarrhea (a), gastric residual > 500 ml (b) and vomiting (c).


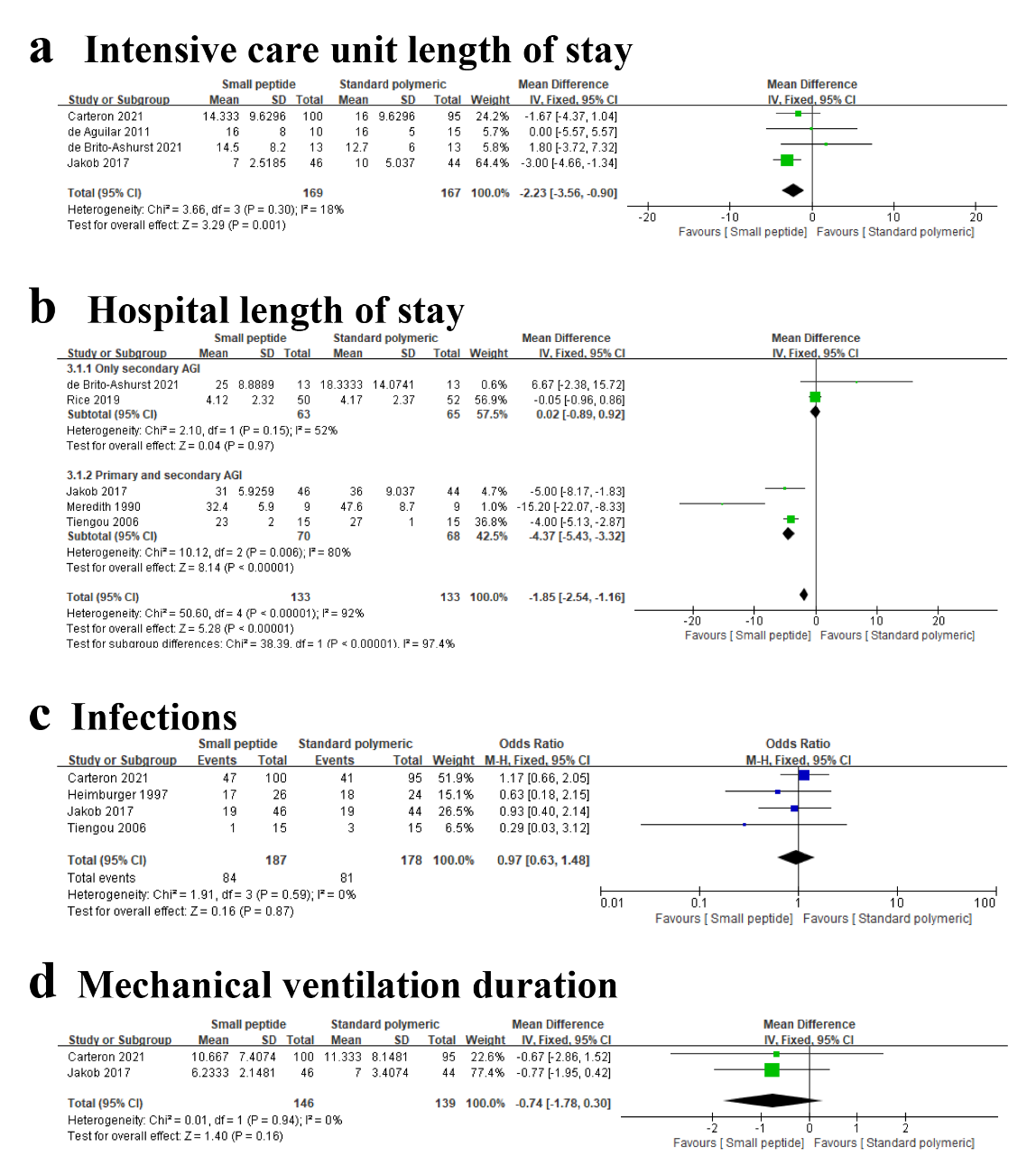


Figure S3. Forest plot for intensive care unit length of stay (a), hospital length of stay (b), infections (c) and mechanical ventilation duration (d).


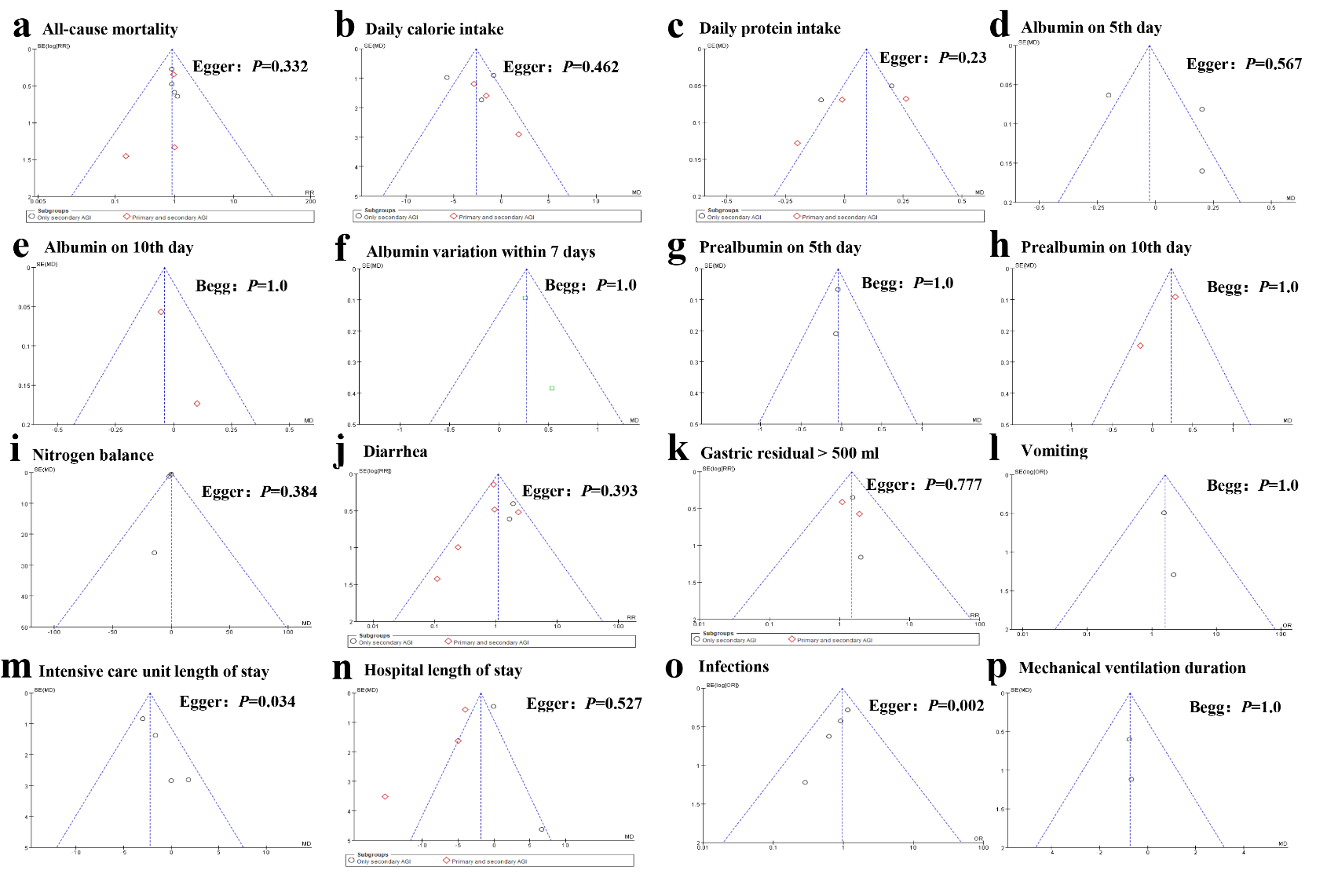


Figure S4. Funnel plots for all-cause mortality (a), daily calorie intake (b), daily protein intake (c), albumin on the 5th day (d), albumin on the 10th day (e), albumin variation within 7 days (f), prealbumin on the 5th day (g), prealbumin on the 10th day (h), nitrogen balance (i), diarrhea (j), gastric residual > 500 ml (k), vomiting (l), intensive care unit length of stay (m), hospital length of stay (n), infections (o) and diarrhea (p).


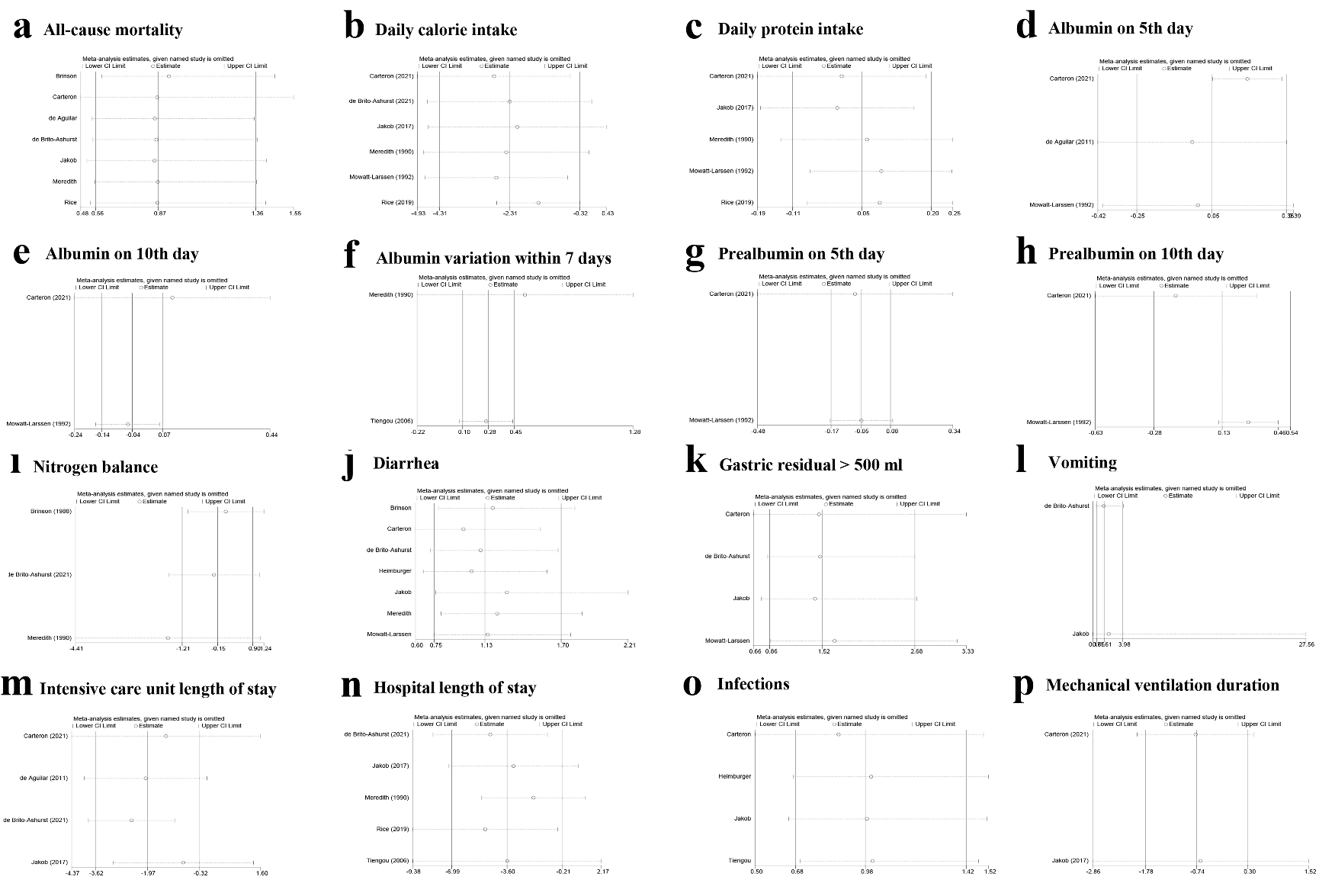


Figure S5. Sensitivity analyses for all-cause mortality (a), daily calorie intake (b), daily protein intake (c), albumin on the 5th day (d), albumin on the 10th day (e), albumin variation within 7 days (f), prealbumin on the 5th day (g), prealbumin on the 10th day (h), nitrogen balance (i), diarrhea (j), gastric residual > 500 ml (k), vomiting (l), intensive care unit length of stay (m), hospital length of stay (n), infections (o) and diarrhea (p).
